# Regulation of renal nitric oxide and eNOS/iNOS expression by tadalafil participates in the mitigation of amphotericin B–induced renal injury: Down-regulation of NF-κB/iNOS/caspase-3 signaling

**DOI:** 10.1007/s00210-023-02787-w

**Published:** 2023-10-28

**Authors:** Doaa M. Abdel-Rahman, Basim Anwar Shehata Messiha, Fares E.M. Ali, Amany A. Azouz

**Affiliations:** 1 Present Address: Department of Pharmacology & Toxicology, Faculty of Pharmacy, Beni-Suef University, Beni-Suef, 62514 Egypt; 2 Department of Pharmacology & Toxicology, Faculty of Pharmacy, Al-Azhar University, Assiut Branch, Assiut, 71524 Egypt

**Keywords:** Amphotericin B–induced AKI, Tadalafil, NO, eNOS/iNOS, Nuclear NF-κB p65, Cleaved caspase-3

## Abstract

Amphotericin B (AmB)–induced acute kidney injury (AKI) is a common health problem having an undesirable impact on its urgent therapeutic utility for fatal systemic fungal infections. Tadalafil (TAD), a phosphodiesterase-5 (PDE-5) inhibitor, has been observed to have a wide range of pharmacological actions, including nephroprotection. The study’s objective was to examine the possible underlying protective mechanism of TAD against AmB-induced nephrotoxicity. Experimentally, animals were divided randomly into four groups: control, TAD (5 mg/kg/day; p.o.), AmB (18.5 mg/kg/day; i.p.), and TAD+AmB groups. Sera and tissue samples were processed for biochemical, molecular, and histological analyses. The biochemical investigations showed that TAD significantly ameliorated the increase of kidney function biomarkers (creatinine, urea, CysC, KIM-1) in serum, renal nitric oxide (NO), lipid peroxidation (MDA), and inflammatory cytokines (TNF-α, IL-6) in AmB-treated rats. Meanwhile, TAD significantly retarded AmB-induced decrease in serum magnesium, sodium, potassium, and renal glutathione content. Molecular analysis revealed that TAD reduced AmB-induced imbalance in the protein expression of eNOS/iNOS, which explains its regulatory effect on renal NO content. These results were also supported by the down-regulation of nuclear NF-κB p65 and cleaved caspase-3 protein expressions, as well as the improvement of histological features by TAD in AmB-treated rats. Therefore, it can be suggested that TAD could be a promising candidate for renoprotection against AmB-induced AKI. That could be partly attributed to its regulatory effect on renal eNOS/iNOS balance and NO, the inhibition of NF-κB p65 nuclear translocation, its downstream inflammatory cytokines and iNOS, and ultimately the inhibition of caspase-3-induced renal apoptosis.

**Supplementary Information:**

The online version contains supplementary material available at 10.1007/s00210-023-02787-w.

## Introduction

Amphotericin B (AmB) is a broad-spectrum antifungal that remains the major fungicidal drug for treating serious systemic mycotic infections (Obayes, 2020). Despite its therapeutic benefits, AmB has several adverse effects on the kidney that limit treatment continuation (Goyal et al., 2020, Salehzadeh et al., 2020, Gursoy et al., 2021).

The nephrotoxic effect of AmB has been attributed to its ability to produce renal tubular injury, electrolyte imbalance, renal vasoconstriction, oxidative stress, and inflammatory response (Fanos and Cataldi, 2000, Laniado-Laborín and Cabrales-Vargas, 2009). Therapeutically, AmB kills fungi by binding to ergosterol in the cell membrane resulting in pore formation and imbalance of K^+^, Na^+^, and Mg^2+^ ions. Similarly, human renal tubular injury and electrolyte imbalance occur with AmB administration (Berto and Dalzochio, 2021, Cavassin et al., 2021). Additionally, AmB has been reported to stimulate nitric oxide (NO) synthesis through the inflammatory cytokine-activated inducible nitric oxide synthase (iNOS) (Mozaffarian et al., 1997, Trajkovic et al., 2001).

Among many studies, only saline loading and liposomal AmB have been shown to prevent or decrease AmB nephrotoxicity. However, both modalities have adverse effects like worsening of congestive heart failure and cirrhosis by saline intake and the high cost of lipid formulations associated also with some nephrotoxic effects and imbalance of electrolytes (Karimzadeh et al., 2020, Panahi-Shokouh et al., 2020). That necessitates the search for novel modalities having pronounced efficacy and safety.

Phosphodiesterase (PDE) enzymes are responsible for the regulation of cAMP and cGMP levels in the cell through their degradation to AMP and GMP (Tzoumas et al., 2020). PDE-5 is one of the PDE subtypes expressed in various smooth muscle cells involving the kidneys (renal vessels, glomeruli, medullary collecting tubules, cortical tubules) (Coskuner and Ozkan, 2021). It has been reported that PDE-5 inhibitors could protect against kidney injury of various etiologies (Afsar et al., 2015, Coskuner and Ozkan, 2021). PDE-5 inhibitors improve renal functions by different mechanisms, including vasodilation and increased renal blood flow via NO/cGMP signaling, inhibition of oxidative stress, inflammation, and renal cell apoptosis (Georgiadis et al., 2020, Coskuner and Ozkan, 2021).

Tadalafil (TAD) is commonly utilized for erectile dysfunction and pulmonary hypertension therapy as a PDE-5 inhibitor which leads to the accumulation of cGMP, an effector second messenger that mediates the vasodilator effect of NO (Grossman, 2022, Maron et al., 2022). In addition, the regulatory effects of TAD on NO production through iNOS and endothelial NOS (eNOS) enzymes have been reported to participate in attenuation of inflammatory signaling and cytokine production (Azouz et al., 2020). Interestingly, the protective effects of TAD against renal ischemia/reperfusion injury, cisplatin, and gentamicin nephrotoxicity have been elucidated by different studies (Adeneye and Benebo, 2016, Maruyama et al., 2022, Mohammed et al., 2022). Therefore, we designed our study to investigate for the first time the possible underlying defensive mechanism of TAD against AmB-induced AKI in rats.

## Materials and methods

### Drugs

The used vials of AmB were obtained from Cipla LTD. (INDIA). Tadalafil was obtained from Lilly (USA).

### Animals

Male Wistar rats at a weight of 200–250 g were purchased from Nahda University Animal House. The rats were kept at a temperature of 25 ± 2 °C, a humidity of 60 ± 10%, and 12-h light/dark cycles. The experimental procedures were approved by the Institutional Animal Care and Use Committee at Beni-Suef University (IACUC-BSU, 019-85) and followed the National Institutes of Health protocol for the care and use of laboratory animals.

### Experimental procedures and sample processing

Rats were divided into 4 groups (5 rats/group). Control group received the vehicle (0.5% CMC; p.o.) only. TAD group received TAD at a dose of 5 mg/kg/day p.o. for 14 days (Iordache et al., 2020). AmB group received AmB at a dose of 18.5 mg/kg/day i.p. from the 8th day of the experiment (Azouz et al., 2023). TAD+AmB group received TAD at a dose of 5 mg/kg/day p.o. for 14 days, and AmB at a dose of 18.5 mg/kg/day i.p. from the 8th day of the experiment.

Samples (blood and kidneys) were taken 24 h after the last dose. The serum samples were frozen at – 20 °C until creatinine, urea, cystatin C (CysC), kidney injury molecule-1 (KIM-1), sodium, potassium, and magnesium levels were assessed. The renal somatic index (RSI) was calculated after removing the kidneys, washing them in phosphate buffer saline, letting them air-dry between layers of filter paper, and then weighing them. That was followed by rapid snap-freezing of kidney samples in liquid nitrogen. Part of each sample was homogenized in phosphate buffer for estimation of NO, malondialdehyde (MDA), reduced glutathione (GSH), tumor necrosis factor-alpha (TNF-α), and interleukin-6 (IL-6) levels. The Western blotting samples were frozen at − 20 °C in a lysis solution containing protease inhibitor. For histological analysis, kidney tissue was preserved in 10% formal saline at room temperature.

### Calculation of the RSI

Kidney weight has been previously reported as a sensitive indicator of nephrotoxicity, where nephrotoxicants increase absolute and relative kidney weight (Kluwe, 1981). RSI was determined in our study following the previously reported method of Abdelrahman and Abdelmageed (2020). Briefly, the body weight of animals and the weight of the left kidney were recorded for each animal then used to calculate RSI as follows:

Renal somatic index (%) = (kidney weight (g)/ body weight (g)) × 100

### Assessment of kidney function

Serum creatinine and urea levels, two common measures of renal function, were determined using kits of SPINREACT (Spain), CAT#: MD1001111 and MI41041, respectively, whereas serum CysC and KIM-1 were estimated via ELISA kits from Glory Science Co., Ltd, USA (CAT#: 30321 for CysC, 11138 for KIM-1), following the guidelines of the manufacturer.

### Estimation of serum electrolyte levels

We used BioMed colorimetric kits to measure sodium (CAT#: SOD100100), potassium (CAT#: POT100100), and magnesium (CAT#: MG122120) in the serum, following the manufacturer’s guidelines.

### Determination of kidney inflammatory biomarkers

Kidney tissue homogenates were analyzed via ELISA kits for IL-6 (CAT#: 95053; Glory Science, USA) and TNF-α (CAT#: 30635; Glory Science, USA), according to the manufacturer’s instructions.

### Estimation of renal oxidant/antioxidant balance

Spectrophotometric analysis of renal GSH was performed according to Ellman (1959). In summary, the sulfhydryl group of GSH combines with Ellman’s reagent (5,5′-dithiobis(2-nitrobenzoic acid)) at controlled pH to create a yellow color, detected at 412 nm. In addition, the amount of NO in the kidney was determined using its stable metabolite (NO_2_^−^) as a substrate, diazotizing sulfanilamide, and coupling the product with N-(1-naphthyl) ethylenediamine dihydrochloride (NEDD). The resultant azo dye with reddish-purple hue was calorimetrically measured at 540 nm as described by Montgomery and Dymock (1961). In accordance with the method reported by Mihara and Uchiyama (1978), MDA, a byproduct of lipid peroxidation, was quantitatively estimated through the intensity of the color produced upon reaction with thiobarbituric acid in acidic medium at 95 °C for 45 min. The produced pink color was extracted by n-butanol for spectrophotometric analysis at 535 nm and 520 nm.

### Western blot analysis

Under cooled conditions, kidney samples were homogenized in Tris lysis buffer (400 mM NaCl, 0.5% Triton X-100, 50 mM Tris pH 7.4) and protease inhibitor cocktail (Biospes, China) for 30 min (Ali et al., 2018). Tissue remnants were removed using centrifuge at 12,000 rpm, 4 °C for 15 min. The separation of nuclear fraction was performed as described previously (Azouz et al., 2022). Protein concentration in the samples was estimated through Biuret technique (Wang et al., 1996). The proteins (50 μg) were equally resolved via 12.5% SDS-polyacrylamide gel electrophoresis. Semi-dry transfer techniques were utilized to transfer the resolved proteins to a PVDF membrane (Millipore, Biospes, China) (Towbin et al., 1979). For 1 h at room temperature, the membranes were blocked with 5% non-fat milk in TBST buffer before being treated with the primary antibodies overnight at 4 °C: eNOS (Santa Cruz, USA, CAT#: sc-136977), iNOS (Santa Cruz, USA, CAT#: sc-7271), nuclear factor-kappa B p65 (NF-κB p65) (Santa Cruz, USA, CAT#: sc-166182), cleaved caspase-3 (Biospes, China, CAT#: YPA2210), and β-actin (Santa Cruz, USA, CAT#: sc-47778). Afterward, the samples were incubated with the proper alkaline phosphatase-conjugated secondary antibodies at room temperature for the next 2 h. BCIP/NBT Kit was utilized for band detection. Densitometric analysis (ImageJ^®^ software, USA) was applied to measure intensity of the bands and calculate the mounts of the estimated proteins relative to a housekeeping protein (β-actin).

### Histopathological analysis

For 24 h, kidney tissue samples from each group were fixed in 10% formal saline. Afterward, samples were dehydrated in serial dilutions of alcohol, cleaned in xylene, and embedded in paraffin. Hematoxylin and eosin (H&E) stain was applied to paraffin block slices cut at a thickness of around 4.5–5 μm (Suvarna et al., 2018). Scoring of the histological alterations was performed as reported by Abdel-Razek et al. (2020).

### Statistical analysis

To statistically analyze the results, GraphPad Prism version 8 (GraphPad Software Inc., USA) was used. The results were shown as the mean ± standard error (SE). One-way ANOVA and Tukey’s post hoc test were applied to compare between results of different groups, where the difference was recognized at *p* < 0.05.

## Results

### TAD restored the normal RSI

**Fig. 1:**
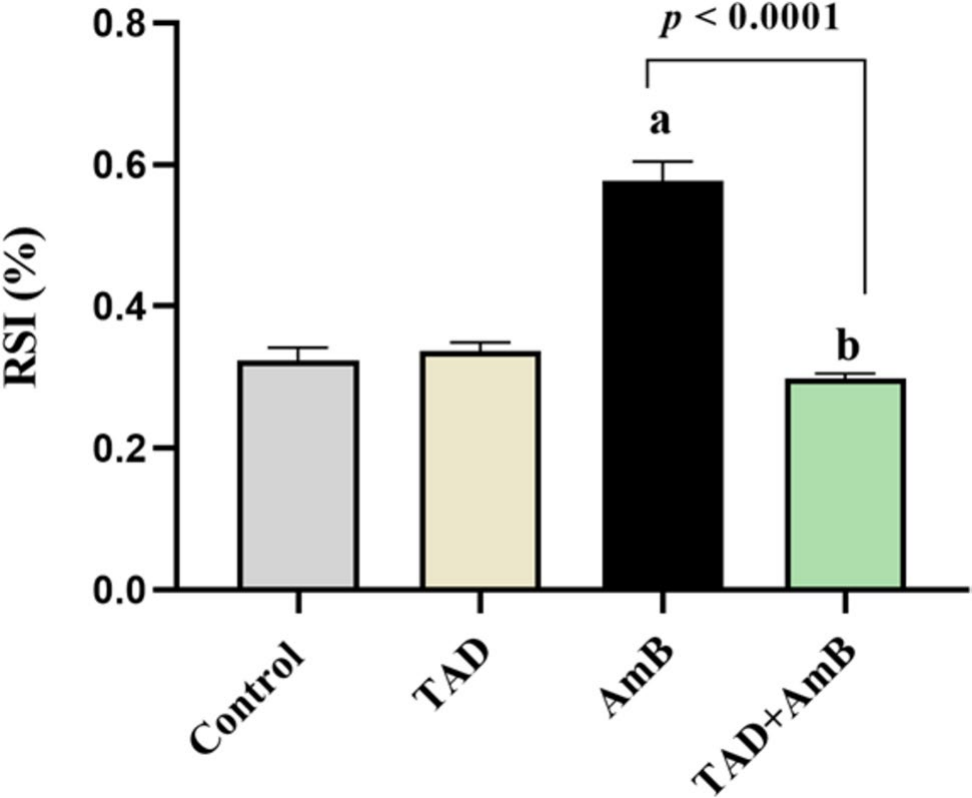
Effect of TAD on RSI in AmB-intoxicated rats. Daily injection of AmB for 1 week significantly increased RSI, while TAD pretreatment with AmB exhibited normalization of RSI. Each bar in the graph represents the mean ± SE (*n* = 5). One-way ANOVA and then Tukey’s test were applied for investigation of the significant difference between groups. ^a^ Significance from control, ^b^ Significance from AmB at *p* < 0.05

Renal somatic index was significantly increased by 78.34% in rats treated with AmB in comparison with the control group. Interestingly, TAD treatment produced markedly reduced RSI by 48.36% at *p* < 0.0001 compared to AmB group (Fig. 1).

### TAD ameliorated AmB-induced kidney dysfunction

**Fig. 2:**
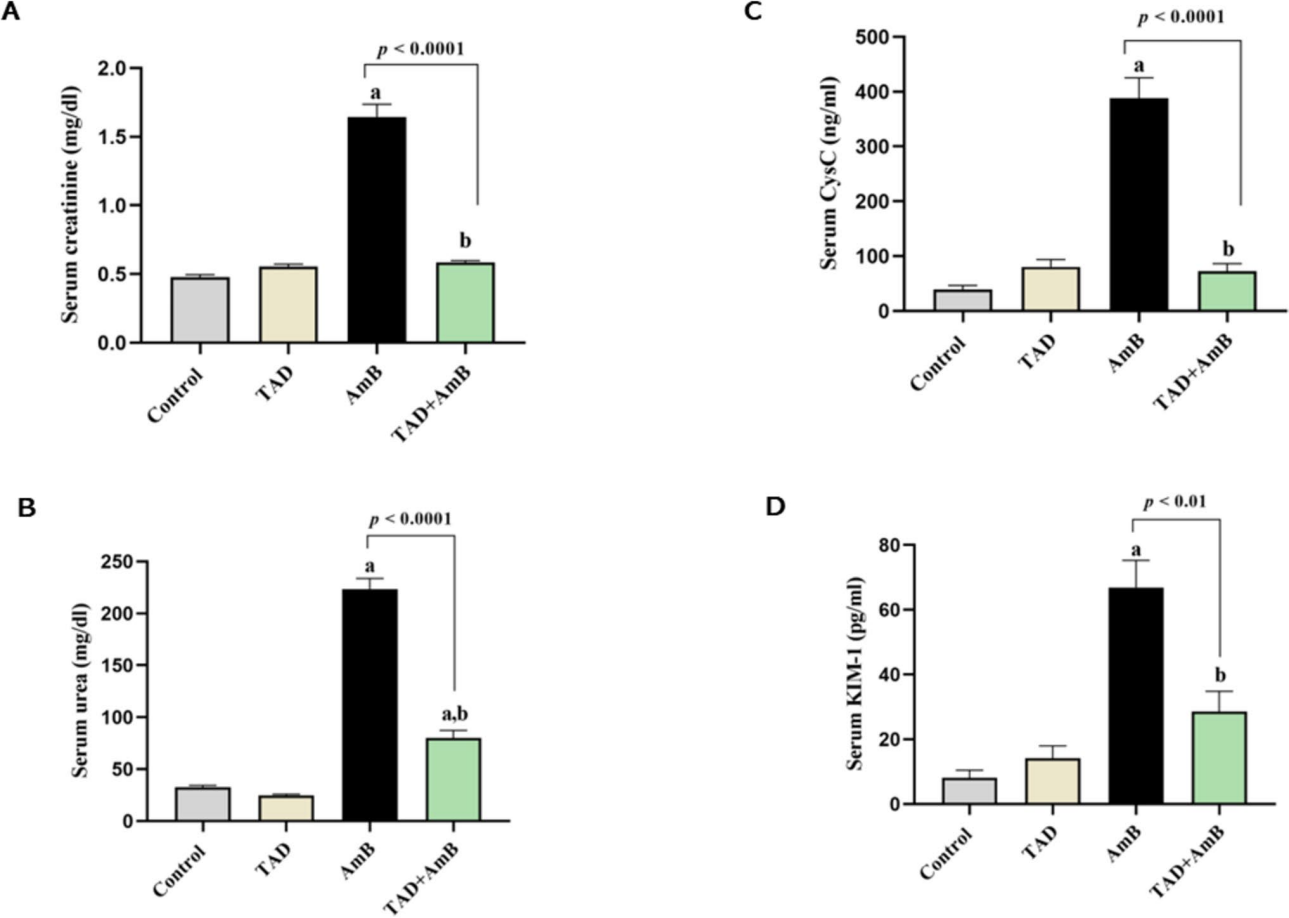
Ameliorative effect of TAD on kidney function deterioration induced by AmB in rats. **A** Serum creatinine, **B** urea, **C** CysC, and **D** KIM-1 levels. Daily injection of AmB for 1 week significantly increased the serum levels of creatinine, urea, CysC, and KIM-1, while TAD pretreatment markedly reduced the deterioration of these markers and so improved the functions of the kidney. Each bar in the graph represents the mean ± SE (*n* = 5). One-way ANOVA and then Tukey’s test were applied for investigation of the significant difference between groups. ^a^ Significance from control, ^b^ Significance from AmB at *p* < 0.05

Compared to the control group, the serum creatinine, urea, CysC, and KIM-1 increased by 2.44-, 5.90-, 8.96-, and 7.22-fold, respectively, indicating that AmB promoted renal nephrotoxicity. However, pretreatment with TAD markedly improved kidney function compared to the AmB control group. This effect was demonstrated by a significant decrease in creatinine, urea, CysC, and KIM-1 levels by 64.48% (*p* < 0.0001), 64.19% (*p* < 0.0001), 81.34% (*p* < 0.0001), and 57.38% (*p* < 0.01), respectively (Fig. 2A–D).

### TAD retarded AmB-induced serum electrolytes imbalance

**Fig. 3:**
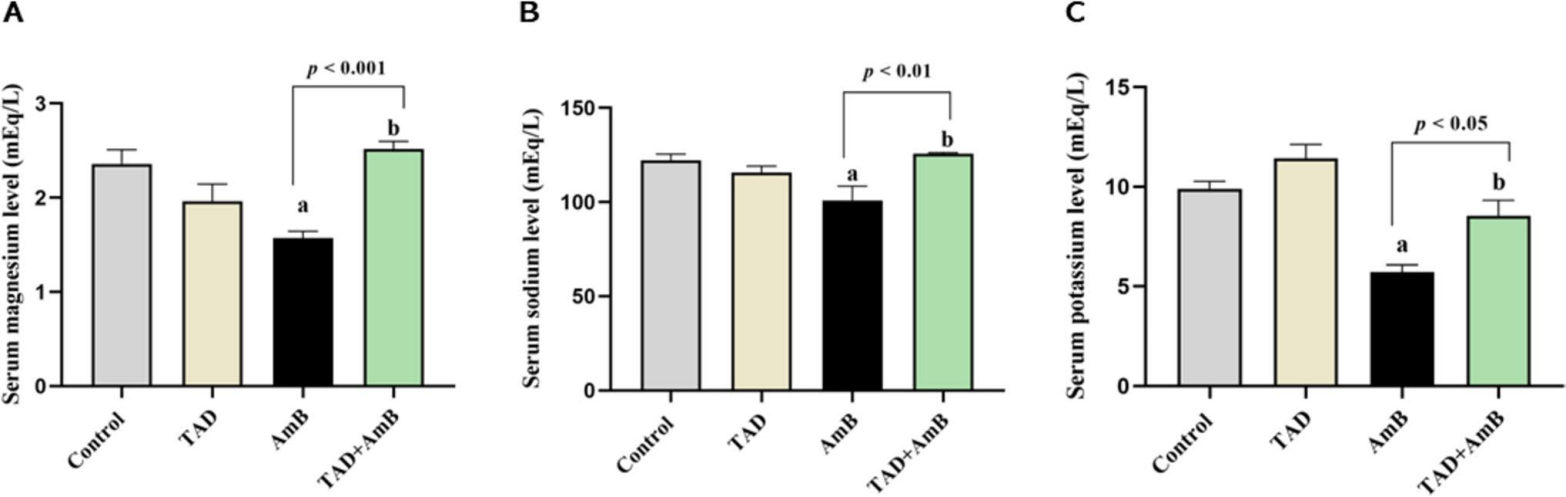
Inhibitory effect of TAD on AmB-induced serum electrolytes imbalance. **A** Serum magnesium, **B** serum sodium, and **C** serum potassium levels. Daily injection of AmB for 1 week significantly increased the serum levels of magnesium, sodium, and potassium, while TAD pretreatment protected against electrolyte imbalance and showed normal levels of serum electrolytes. Each bar in the graph represents the mean ± SE (*n* = 5). One-way ANOVA and then Tukey’s test were applied for investigation of the significant difference between groups. ^a^ Significance from control, ^b^ Significance from AmB at *p* < 0.05

Furthermore, we examined the effect of TAD on the electrolyte disturbance induced by AmB administration. AmB induced serum electrolytes imbalance indicated by the reduction of magnesium, sodium, and potassium levels to 33.45%, 17.35%, and 42.24%, respectively, in comparison with the control rats. Conversely, pretreatment with TAD preserved serum electrolyte levels by 60.45% (*p* < 0.001), 24.36% (*p* < 0.01), and 49.39% (*p* < 0.05), respectively, in comparison with AmB group (Fig. 3A, B, C).

### TAD abrogated AmB-induced renal histopathological changes

**Fig. 4:**
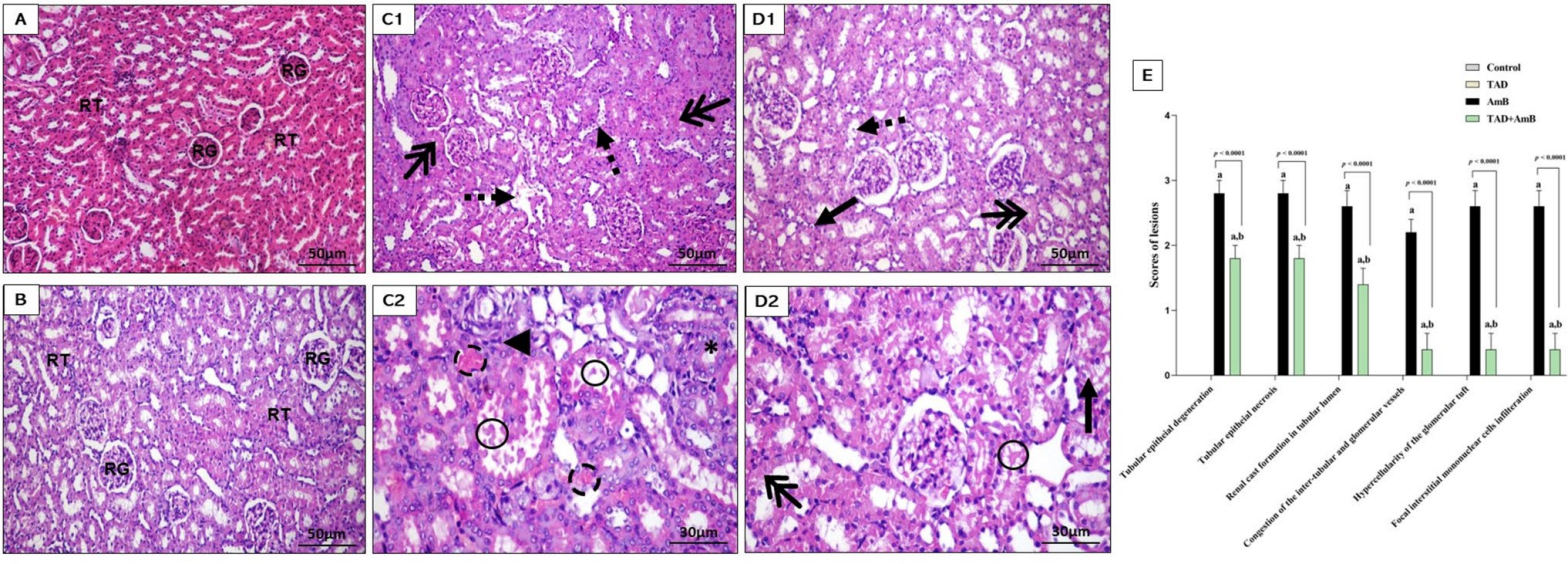
Ameliorative effect of TAD on renal histopathological changes induced by AmB in rats. Microscopic images of kidney sections from the **A** control group and **B** TAD group showing the normal histological structure of renal glomeruli (RG) and renal tubules (RT). **C1** Sections from AmB group showing marked desquamation (dashed arrow), nuclear pyknosis and necrosis (double-headed arrow) of the tubular epithelial linings, **C2** granular cast (circle) formation in the tubular lumen, nuclear hyperchromasia of some tubular epithelium (asterisk), congestion of interstitial vessels (dashed circle), few inflammatory cells infiltration (arrow head), and necrobiotic changes of the tubular epithelial linings. On the other hand, **D1** sections from TAD+AmB group showing mild degree of tubular epithelial lining degeneration (arrow), necrosis (double-headed arrow), and few desquamation (dashed arrow), **D2** moderate vacuolar degeneration (arrow), necrosis (double-headed arrow), and some desquamation of the renal tubular linings with early cast formation in the lumen of some tubules (circle). **E** Scoring of the histopathological lesions showing marked alleviation of the histological alterations by TAD administration to AmB-intoxicated rats

We confirmed our biochemical results by evaluating TAD effect on the renal histological alterations induced by AmB. Kidney sections from control and TAD groups showed the normal histological structure of glomeruli and tubules (Fig. 4A, B). As excepted, necrobiotic changes of the tubular epithelium, including marked desquamation, nuclear pyknosis, and necrosis of the tubular epithelial linings were induced by injection of AmB (18.5 mg/kg) daily for 1 week. Also, granular cast formation in the tubular lumen, congestion of interstitial and glomerular vessels, and inflammatory cell infiltration were observed in sections of AmB-intoxicated rats (Fig. 4 C1, C2). Interestingly, pretreatment with TAD (5 mg/kg) ameliorated the histological changes in the kidney of AmB-intoxicated rats, as indicated by moderate tubular epithelial degenerative and necrotic changes, with some desquamated cells and scattered cast formation (Fig. 4 D1, D2). That was evidenced by the significant reduction (*p* < 0.0001) of histological alteration scoring in TAD+AmB group (Fig. 4E).

### TAD regulated eNOS/iNOS expression and NO content in the kidney after AmB challenge

**Fig. 5:**
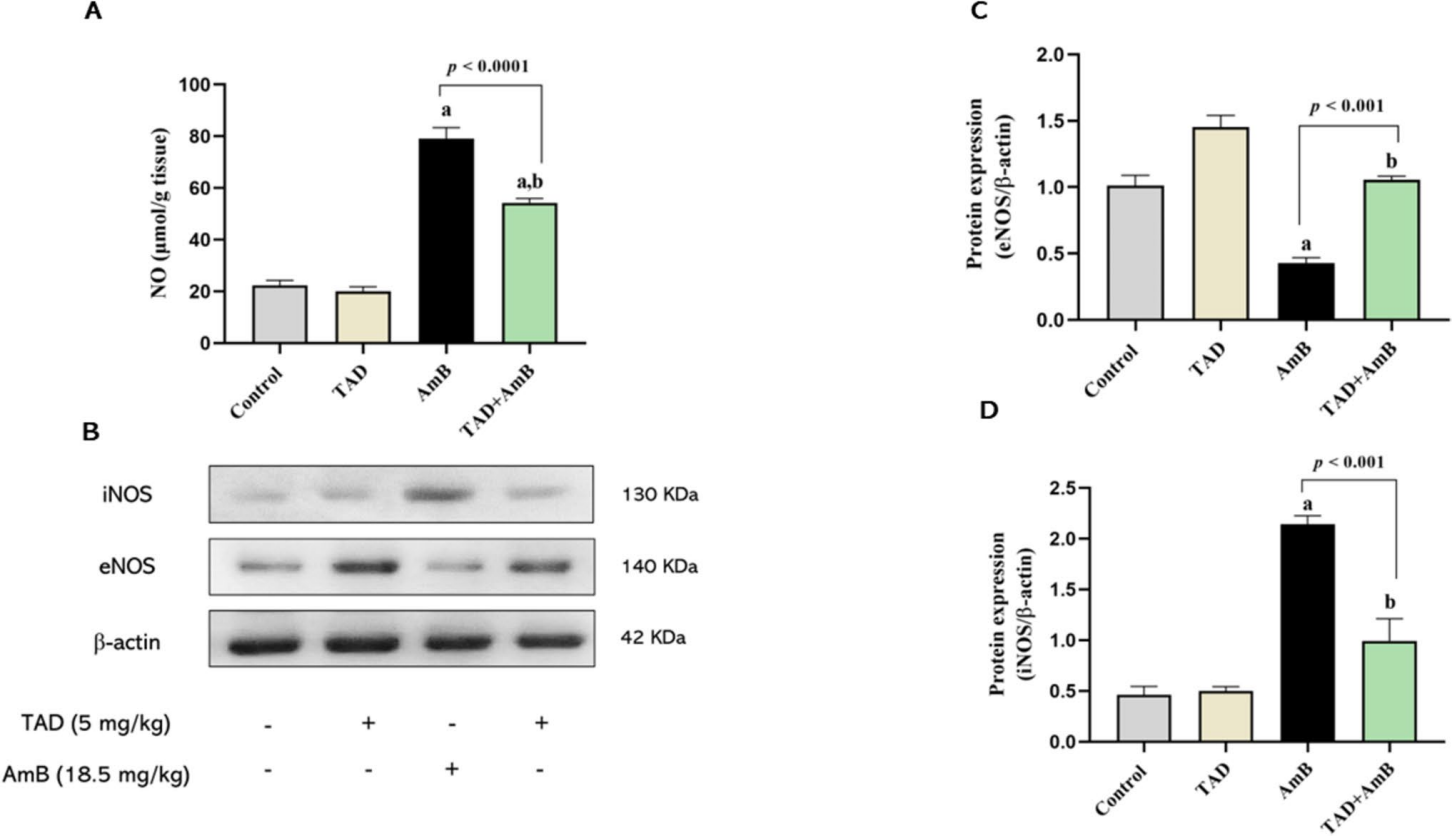
Regulatory effect of TAD on renal NO content and eNOS/iNOS expression in AmB-intoxicated rats. **A** Renal NO content was significantly elevated by AmB, while TAD pretreatment markedly ameliorated the elevation of NO in AmB-intoxicated rats (*n* = 5). **B** Western blots for eNOS and iNOS protein expression. **C**, **D** Graphical presentation of the changes in eNOS and iNOS protein expression, elucidating the regulatory effects of TAD on eNOS/iNOS expression in AmB-intoxicated rats (*n* = 3). Each bar in the graph represents the mean ± SE. One-way ANOVA and then Tukey’s test were applied for investigation of the significant difference between groups. ^a^ Significance from control, ^b^ Significance from AmB at *p* < 0.05

Moreover, we examined the role of eNOS/iNOS balance and NO in the protective mechanism of TAD against AmB nephrotoxicity. By comparison to normal control, injection of rats with AmB significantly up-regulated iNOS expression and NO levels (3.63- and 2.54-fold, respectively), but down-regulated eNOS expression (57.64%). However, pretreatment with TAD significantly reduced iNOS and NO by 53.63% (*p* < 0.001) and 31.32% (*p* < 0.0001), but markedly increased eNOS expression by 1.46-fold (*p* < 0.001) compared to AmB-treated rats (Fig. 5A–D).

### TAD retarded AmB-induced oxidative damage and reinforced antioxidant defense in the kidney

**Fig. 6:**
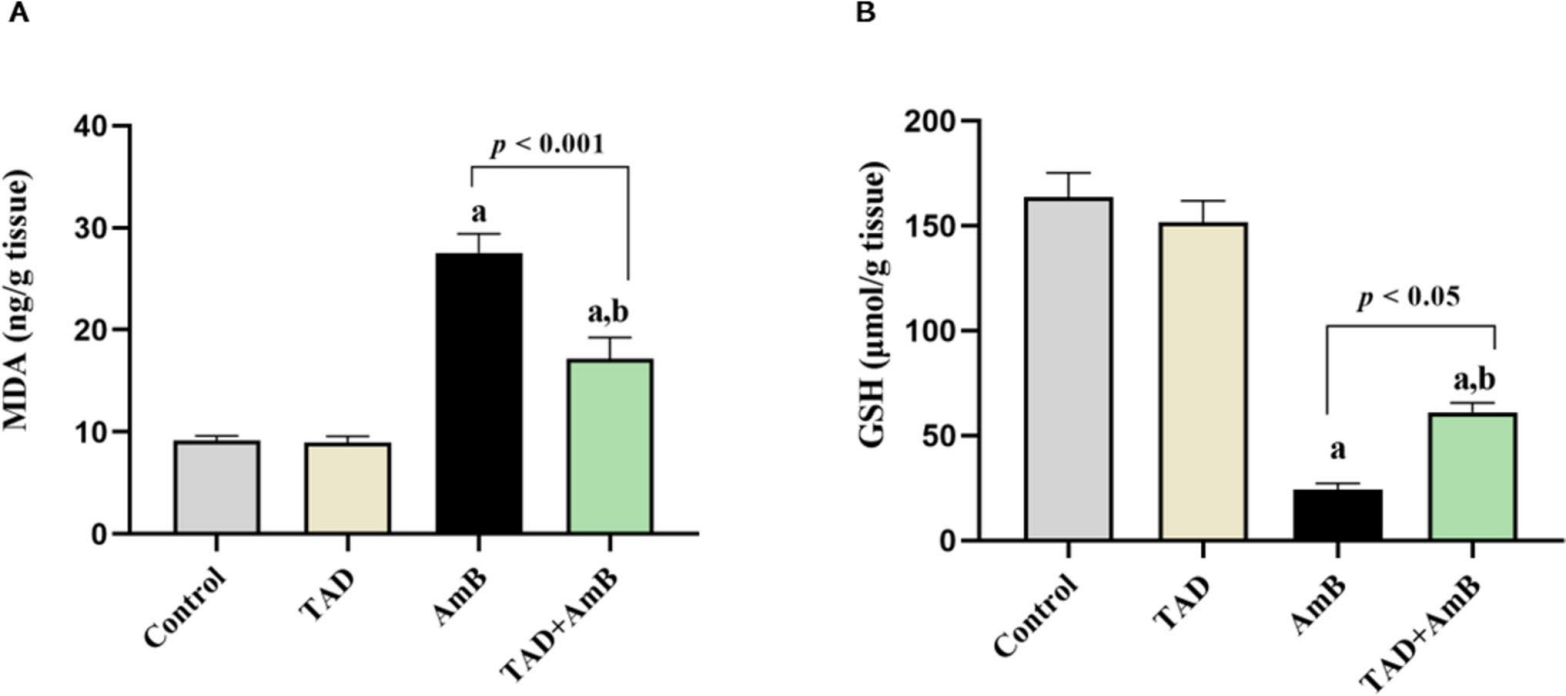
Mitigation of oxidative stress by TAD in AmB-intoxicated rats. Renal A MDA and B GSH contents. AmB-treated rats showed significantly increased renal content of the lipid peroxidation marker MDA, while GSH content was reduced. On the other hand, TAD pretreatment to AmB-intoxicated rats ameliorated the increase of MDA and the reduction of GSH content. Each bar in the graph represents the mean ± SE (*n* = 5). One-way ANOVA and then Tukey’s test were applied for investigation of the significant difference between groups. ^a^ Significance from control, ^b^ Significance from AmB at *p* < 0.05

Renal intoxication with AmB was accompanied by oxidative injury. That was indicated by increased renal MDA content by 2-fold, in addition to significantly declined GSH content by 85.17%, upon comparison to the control group. In contrast, TAD pretreatment mitigated oxidative stress evoked by AmB as evidenced by the considerably reduced MDA content (37.79%, *p* < 0.001) and the preserved GSH content (1.52-fold, *p* < 0.05) compared to AmB-treated rats (Fig. 6A, B).

### TAD alleviated AmB-induced renal inflammatory response

**Fig. 7:**
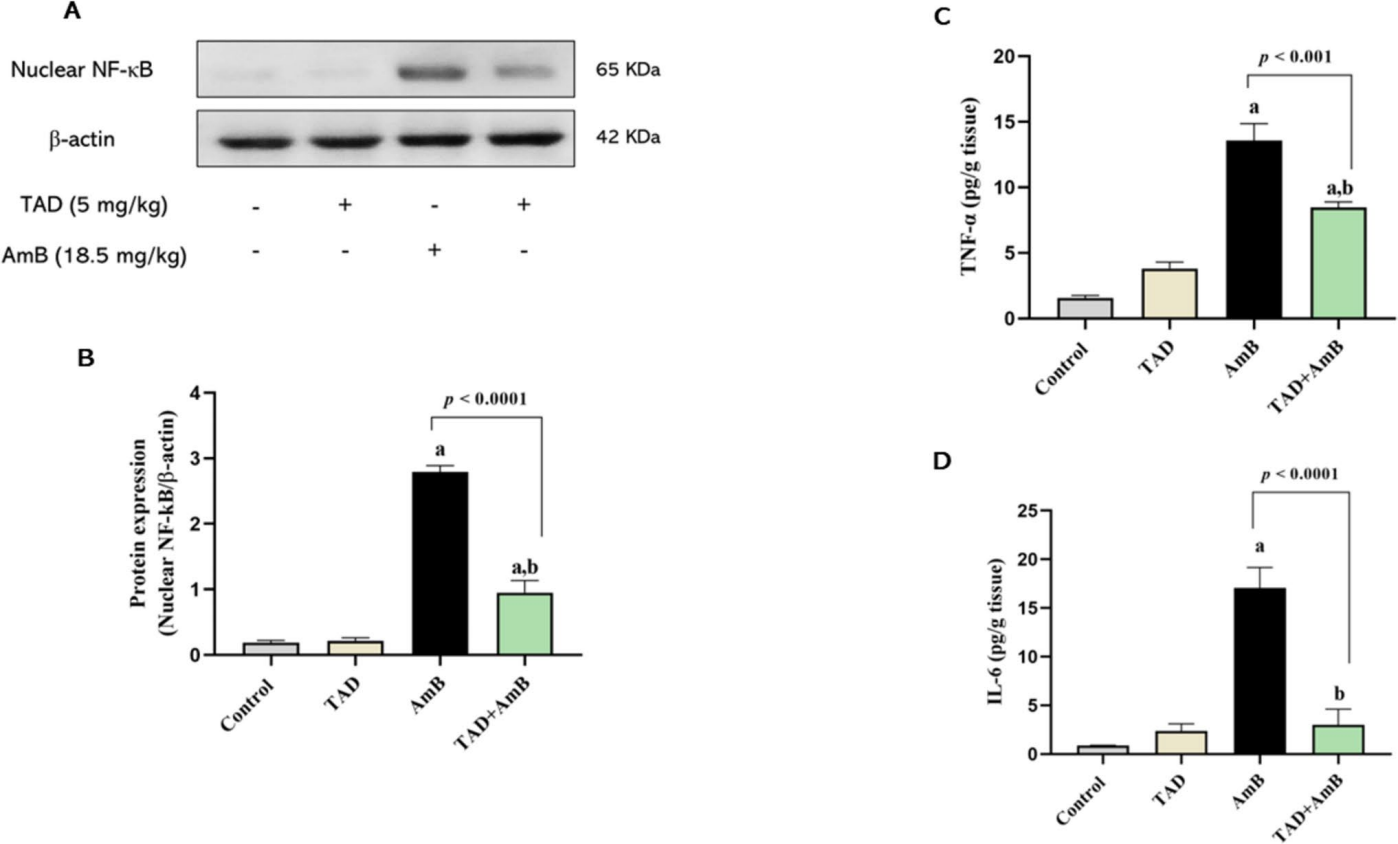
Attenuation of the inflammatory response by TAD in AmB-intoxicated rats. **A** Western blot for nuclear NF-κB and **B** graphical presentation of the changes in nuclear NF-κB expression demonstrating the enhanced expression of NF-κB by AmB, while TAD pretreatment attenuated the effect of AmB. That was evident by reduced renal content of TNF-α (**C**) and IL-6 (**D**) by TAD in AmB-treated rats. Each bar in the graph represents the mean ± SE (*n* = 5). One-way ANOVA and then Tukey’s test were applied for investigation of the significant difference between groups. ^a^ Significance from control, ^b^ Significance from AmB at *p* < 0.05

The involvement of TAD anti-inflammatory properties in the mitigation of AmB-induced renal inflammatory response was assessed through NF-κB/TNF-α signaling. Compared to normal control rats, AmB injection showed up-regulation of nuclear NF-κB protein expression (13.81-fold). However, pretreatment with TAD significantly declined renal nuclear NF-κB by 65.92% (*p* < 0.0001), referring to its anti-inflammatory effect. The up-regulation of TNF-α and IL-6 (7.67- and 18.94-fold, respectively) by AmB also demonstrated the acute inflammatory response generated by AmB, which is considered the first stage in the pathophysiology of its nephrotoxicity. TAD pretreatment significantly reduced renal TNF-α and IL-6 by 37.73% (*p* < 0.001) and 82.35% (*p* < 0.0001), respectively, thus illustrating its anti-inflammatory effect (Fig. 7A–D).

### TAD down-regulated cleaved caspase-3 expression in the kidney after AmB challenge

**Fig. 8:**
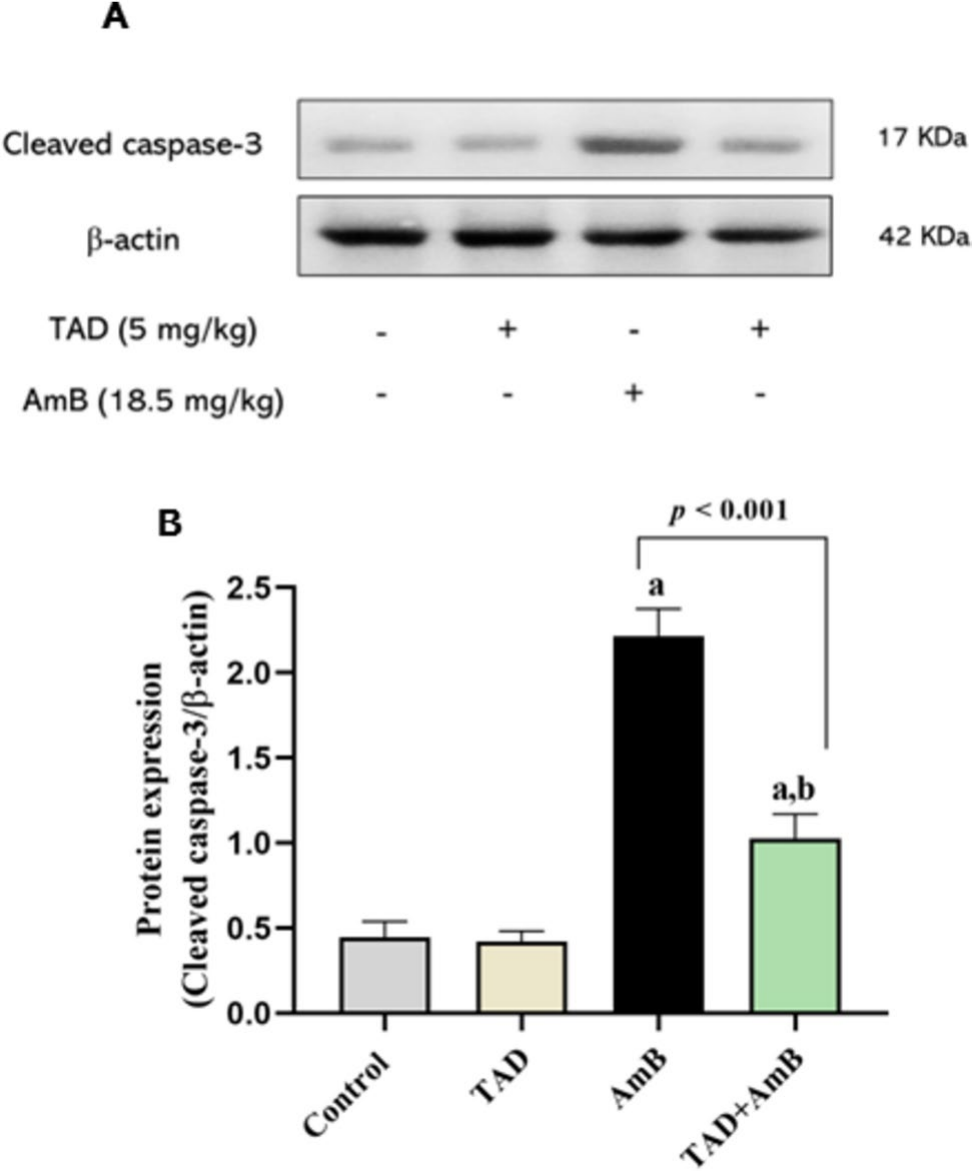
Down-regulation of cleaved caspase-3 expression by TAD in AmB-intoxicated rats. **A** Western blot for cleaved caspase-3 and **B** graphical presentation of the changes in cleaved caspase-3 expression demonstrating enhanced expression of cleaved caspase-3 by AmB, while TAD pretreatment attenuated the effect of AmB. Each bar in the graph represents the mean ± SE (*n* = 3). One-way ANOVA and then Tukey’s test were applied for investigation of the significant difference between groups. ^a^ Significance from control, ^b^ Significance from AmB at *p* < 0.05

Finally, the effect of TAD on AmB-induced renal apoptosis was evaluated. AmB enhanced the activation of the apoptotic effector caspase-3 as indicated by the significantly increased protein expression of cleaved caspase-3 in renal tissue by 3.97-fold compared to the normal control group, whereas administration of TAD down-regulated cleaved caspase-3 expression by 53.68% (*p* < 0.001) in AmB-treated rats, indicating the antiapoptotic effect afforded by TAD (Fig. 8).

## Discussion

The nephrotoxic effect of AmB is attained by its deleterious effects on membrane permeability, reduction in renal blood flow, and glomerular filtration rate, leading eventually to renal injury (Ambreen et al., 2020, Adedeji et al., 2021). Therefore, we investigated the potential protective effect of the PDE-5 inhibitor TAD against AmB-induced nephrotoxicity, based on its previously reported effective vasodilator, anti-inflammatory, and antioxidant properties.

In the current study, TAD ameliorated the elevation in RSI, kidney function biomarkers, and serum electrolyte imbalance in AmB-intoxicated rats. In addition, TAD regulated renal NO content via up-regulation of eNOS and down-regulation of iNOS protein expression. It also attenuated the inflammatory response evoked by AmB through down-regulation of NF-κB and its downstream inflammatory cytokines: TNF-α and IL-6. Ultimately, TAD retarded apoptosis of renal cells evidenced by reduced expression of the apoptotic effector cleaved caspase-3 and the histopathological investigation.

Results of our investigations revealed that repeated administration of AmB elevated serum creatinine, urea, CysC, and KIM-1, indicating renal dysfunction. Our results are in accordance with those of previous studies (Liu et al., 2020, Adedeji et al., 2021, Magalhães et al., 2022). AmB has been reported to cause renal tubular damage through renal vasoconstriction due to its direct effect on vascular smooth muscles, resulting in reduced blood flow to the kidney and so decline of glomerular filtration rate (Adedeji et al., 2021). The aforementioned deleterious effects of AmB on the kidney account for the deterioration in kidney function biomarkers.

Amphotericin B administration also produced a significant increase in RSI that could be attributed to the decrease in body weight as reported previously (Tonomura et al., 2009, Udawatte et al., 2020) or the increase in kidney weight by the nephrotoxic effect of AmB (Kluwe, 1981). Kidney weight relative to body weight has been estimated as an indicator of AmB nephrotoxicity, which results in body weight loss and kidney weight gain, and so the relative kidney/body weight ratio, RSI, increases (Boswell et al., 1998).

In addition, AmB repeated administration in our study resulted in hypomagnesemia, hyponatremia, and hypokalemia. This electrolyte imbalance induced by AmB could be strongly attributed to its binding to cholesterol in the renal cell membrane as it does with ergosterol of fungal cells resulting in the formation of pores across the cell membrane, which subsequently disturbs membrane permeability and leads to electrolyte imbalance (Mahajan, 2019, Ambreen et al., 2020, Downes et al., 2020, Abdel-Hafez et al., 2022).

On the other hand, TAD pretreatment improved kidney functions and rebalanced serum electrolytes in AmB-treated rats. These results are in accordance with the previously reported nephroprotective effect of TAD against contrast- and cisplatin-induced nephropathy (Wehaish et al., 2014, Abassi and Armaly, 2015, Adeneye and Benebo, 2016). Besides, TAD preserved normal RSI. In parallel, the effect of TAD in reducing the relative kidney weight has been demonstrated in a rat model of contrast-induced nephropathy, indicating the nephroprotection conferred by TAD (Iordache et al., 2020).

Nitric oxide has important regulatory functions on smooth muscle relaxation, inflammation, and fibrosis through guanylate cyclase/cGMP signaling (Zimmer et al., 2020). On the other side, increased NO could produce harmful effects on the cell by delaying mitochondrial respiration, inhibiting numerous essential enzymes, causing DNA and membrane damage, and releasing iron from FeS complexes (Valdivielso and Blantz, 2002).

Different studies have elucidated that AmB treatment increases NO levels through the stimulation of iNOS expression and activity, which subsequently produces the toxic effect of AmB (Trajkovic et al., 2001, Suschek et al., 2002, Altuntaş et al., 2014). Meanwhile, AmB accumulation in the kidney has been reported to suppress eNOS protein expression and activity after repeated administration, which could increase vasoconstriction and thrombus formation (Suschek et al., 2000). The inducible isoform of NOS, iNOS, is incorporated in the immunological and inflammatory responses, while the constitutive isoform eNOS participates a crucial defensive role in the kidney through vasodilation and regulation of blood pressure (Cinelli et al., 2020). Our results are in accordance with those of previous studies where AmB-treated rats showed enhanced iNOS protein expression in the kidney, while eNOS expression was reduced, demonstrating the source of elevated renal NO content.

The nephrotoxic effect of AmB leads to ischemia and hypoxia in the endothelial and tubular epithelial cells with subsequent stimulation of the inflammatory response, formation of reactive oxygen species (ROS), and suppression of the antioxidant defense resulting in oxidative injury (Schlottfeldt et al., 2015). Oxidative stress and inflammatory cytokines have been reported to induce iNOS expression through activation of the transcription factor NF-κB (Pautz et al., 2010, Cinelli et al., 2020).

In our study, the group treated with TAD along with AmB showed reduced renal NO content, down-regulated iNOS, and up-regulated eNOS protein expression by comparison to AmB group. Parallel to our results, a previous study has demonstrated the protective of TAD agaist gentamicin-induced nephrotoxicity by regulating renal NO content and iNOS/eNOS expression (Mohammed et al., 2022). These regulatory effects of TAD could be partly attributed to its anti-inflammatory and antioxidant properties that attenuate NF-κB/iNOS signaling. In addition, the stimulatory effect of TAD on eNOS could be related to PDE-5 inhibition which preserves endothelial function as previously reported (El-Sayed and Amin, 2015).

The current investigation revealed that AmB injection increased renal lipid peroxidation, as evidenced by the elevation in renal MDA content, while the antioxidant defense declined, as evidenced by the depletion of renal GSH content. Our results align with previous studies that have demonstrated disturbed oxidant/antioxidant balance by AmB in the kidney (Altuntaş et al., 2014, Salehzadeh et al., 2020, Azouz et al., 2023). The excessive generation of ROS induces oxidative damage and disturbs the oxidant/antioxidant balance, resulting in lipid peroxidation, protein oxidation, and DNA damage (Liu et al., 2022). Moreover, it has been reported that elevated ROS and NO levels may worsen cellular damage by reducing intracellular GSH levels and antioxidant capacity (de Pinto et al., 2002, Ozbek et al., 2009).

In our study, pretreatment with TAD showed remarkable attenuation of oxidative damage in AmB-treated rats where renal MDA content was reduced while GSH content was elevated. These results are parallel to those of previous studies elucidating the nephroprotective effect of TAD against contrast-induced nephropathy, diabetic nephropathy, gentamicin-, and cisplatin-induced nephrotoxicity models via its antioxidant properties (Adeneye and Benebo, 2016, Elhawary and Abd Allah, 2017, Iordache et al., 2020, Mohammed et al., 2022).

Interestingly, the increased production of ROS has been reported to stimulate the inflammatory response through p38 mitogen-activated protein kinase (MAPK)/NF-κB signaling. The phosphorylation of the inhibitor of nuclear factor kappa B (IκB) by IκB kinases promotes the dissociation and nuclear translocation of NF-κB, which stimulates the transcription of certain genes involved in the inflammatory response, including iNOS, TNF-α, and IL-6 (Ozbek et al., 2009, Ma et al., 2017). Furthermore, the activation of MAPKs has been reported to promote AmB nephrotoxicity through sodium entry into the cells via membrane pores formed by AmB binding to membrane cholesterol, causing depolarization. That in turn leads to increased intracellular calcium with subsequent activation of calcium-dependent proteases, resulting in cellular structure disruption and cell death (Yano et al., 2009, Downes et al., 2020).

Our results revealed that AmB stimulated the nuclear translocation of the transcription factor NF-κB, and so the pro-inflammatory cytokines TNF-α and IL-6 were up-regulated. In accordance, the stimulatory effect of the antifungal AmB on immune cells via CD14-Toll-like receptors (TLRs)/NF-κB signaling and the subsequent production of inflammatory cytokines have been reported previously (Sau et al., 2003).

On the other side, the anti-inflammatory effect of TAD was evidenced by reduced nuclear expression of NF-κB and the kidney levels of TNF-α and IL-6. Parallel to our results, the anti-inflammatory effect of TAD against potassium dichromate-induced renal injury has been reported with the demonstration of its inhibitory effect on TNF-α (Salama et al., 2016). Similarly, the inhibitory effect of TAD on TNF-α, IL-1β, and its stimulatory effect on IL-10 against renal I/R injury have been reported previously (Carvalho et al., 2015, El-Sisi et al., 2016). In accordance, Li et al. (2020) have demonstrated via Western blotting the inhibitory effect of TAD on the phosphorylation of IκBα/NF-κB in placental and renal tissues of rats with L-NAME-induced pre-eclampsia, as well as the reduced plasma levels and placental and renal mRNA expressions of the pro-inflammatory cytokines TNF-α and IL-6. Besides, PDE-5 inhibition has been elucidated to produce a marked reduction in renal TNF-α and NF-κB levels in diclofenac nephrotoxicity model (Wadie et al., 2021). Therefore, the nephroprotective effects of TAD could be partly explained by its anti-inflammatory properties.

Our Western blot results indicated the activation of caspase-3 in AmB group, which is in accordance with the previously reported apoptotic effect of AmB on the kidney (Varlam et al., 2001, Grossi et al., 2017, Azouz et al., 2023). Interestingly, a previous study has elucidated the role of NO in neutrophil apoptosis. The increased generation of NO through iNOS activation has been reported to stimulate ROS production via NOX2, followed by caspase-8 activation that initiates mitochondrial apoptotic signaling with procaspase-3 cleavage, leading eventually to apoptosis (Dubey et al., 2016). Furthermore, the disturbed balance of iNOS/eNOS has been reported to promote endoplasmic reticulum stress and placental apoptosis in pre-eclampsia (Du et al., 2017).

Otherwise, the antiapoptotic effect of TAD was confirmed in our study by inhibited activation of the apoptotic executioner caspase-3, where the protein expression of cleaved caspase-3 was down-regulated in the TAD+AmB group by comparison to AmB group. These results parallel those of previous studies that have confirmed the antiapoptotic effect of TAD against I/R- and adenine-induced renal failure via reduced caspase-3 expression (El-Sisi et al., 2016, Hamdy et al., 2022). Based on the above-mentioned correlation between iNOS/NO and apoptosis, the antiapoptotic effects of TAD in our study could be attributed to its regulatory effects on eNOS/iNOS and renal NO content.

Furthermore, the histopathological alterations in our study were consistent with the aforementioned deleterious effects of AmB on the kidney and the nephroprotective effects of TAD, which supports our suggestion of the therapeutic benefit of TAD administration with AmB to mitigate its nephrotoxic effect.

## Conclusion

Our findings revealed that TAD inhibited AmB-induced AKI, where TAD guarded against kidney dysfunction and serum electrolyte imbalance. These defensive effects of TAD could be attributed to its regulatory effects on eNOS/iNOS expression and renal NO content. In addition, the suppressor effect of TAD on inflammatory signaling via reduced nuclear translocation of NF-κB and reduced formation of the inflammatory cytokines, TNF-α and IL-6, could partially explain its regulatory effects on iNOS/NO signaling. Ultimately, these effects of TAD could certainly contribute to retarding activation of the apoptotic executioner caspase-3 and reducing renal apoptosis. Thus, it can be suggested that TAD administration with AmB could reduce its nephrotoxic effect and increase the tolerability to multiple-dose administration of AmB in life-threatening systemic fungal infections. Further clinical studies are required to confirm the therapeutic benefits of TAD administration with AmB.

### Supplementary information

ESM 1 (PPTX 10225 kb)

## Data Availability

Data are available from the corresponding author upon reasonable request.
