# Supplementary material for: Regulation of renal nitric oxide and eNOS/iNOS expression by tadalafil participates in the mitigation of amphotericin B–induced renal injury: Down-regulation of NF-κB/iNOS/caspase-3 signaling
Source: Naunyn Schmiedebergs Arch Pharmacol. 2023 Oct 28;397(5):3141–53. doi: 10.1007/s00210-023-02787-w (PMC11074040; doi:10.1007/s00210-023-02787-w)
Supplement: Supplementary file 1 — (PPTX 10225 kb) [file 210_2023_2787_MOESM1_ESM.pptx]

## Slide 1
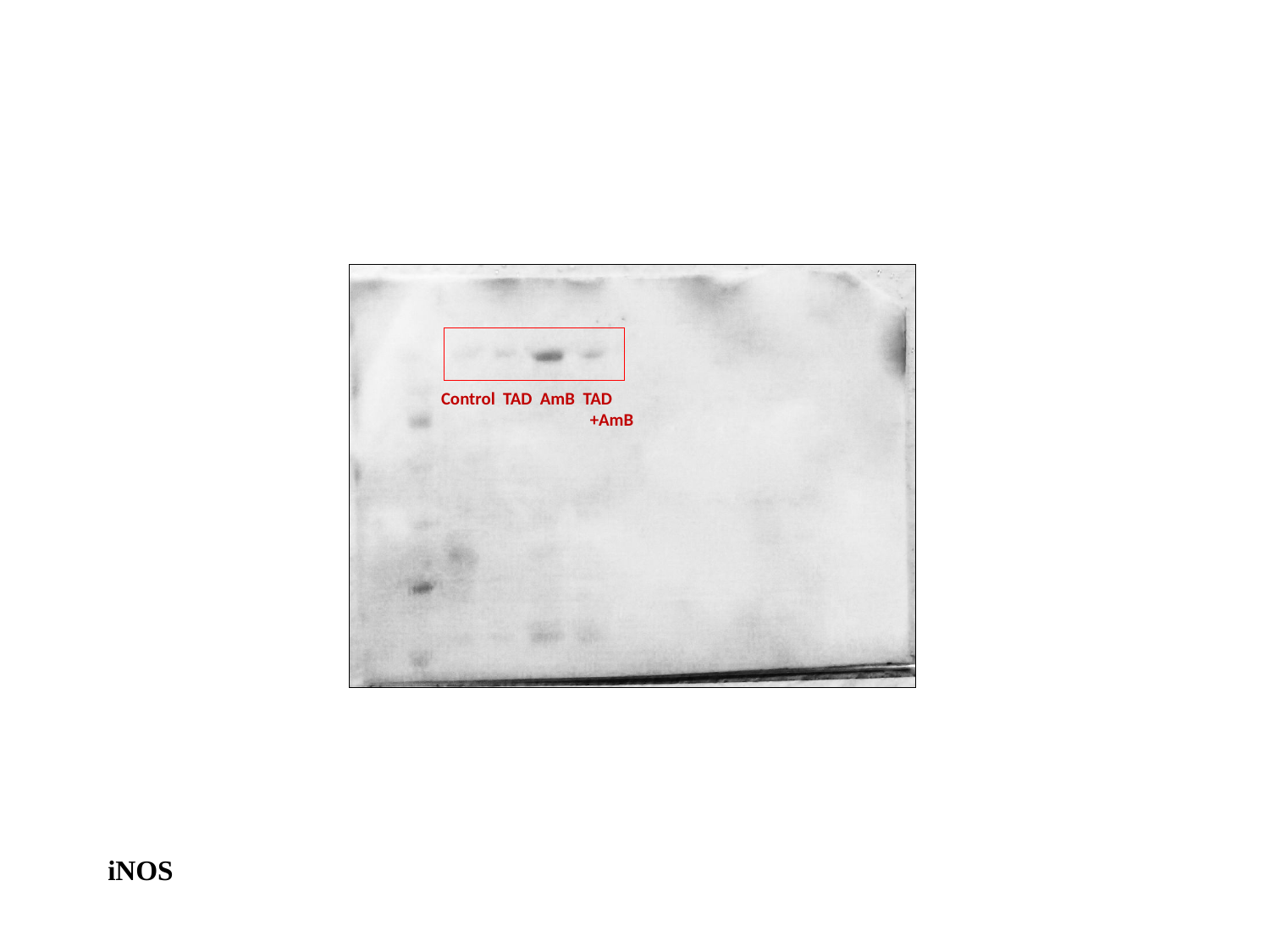

Control TAD AmB TAD
 +AmB
iNOS

## Slide 2
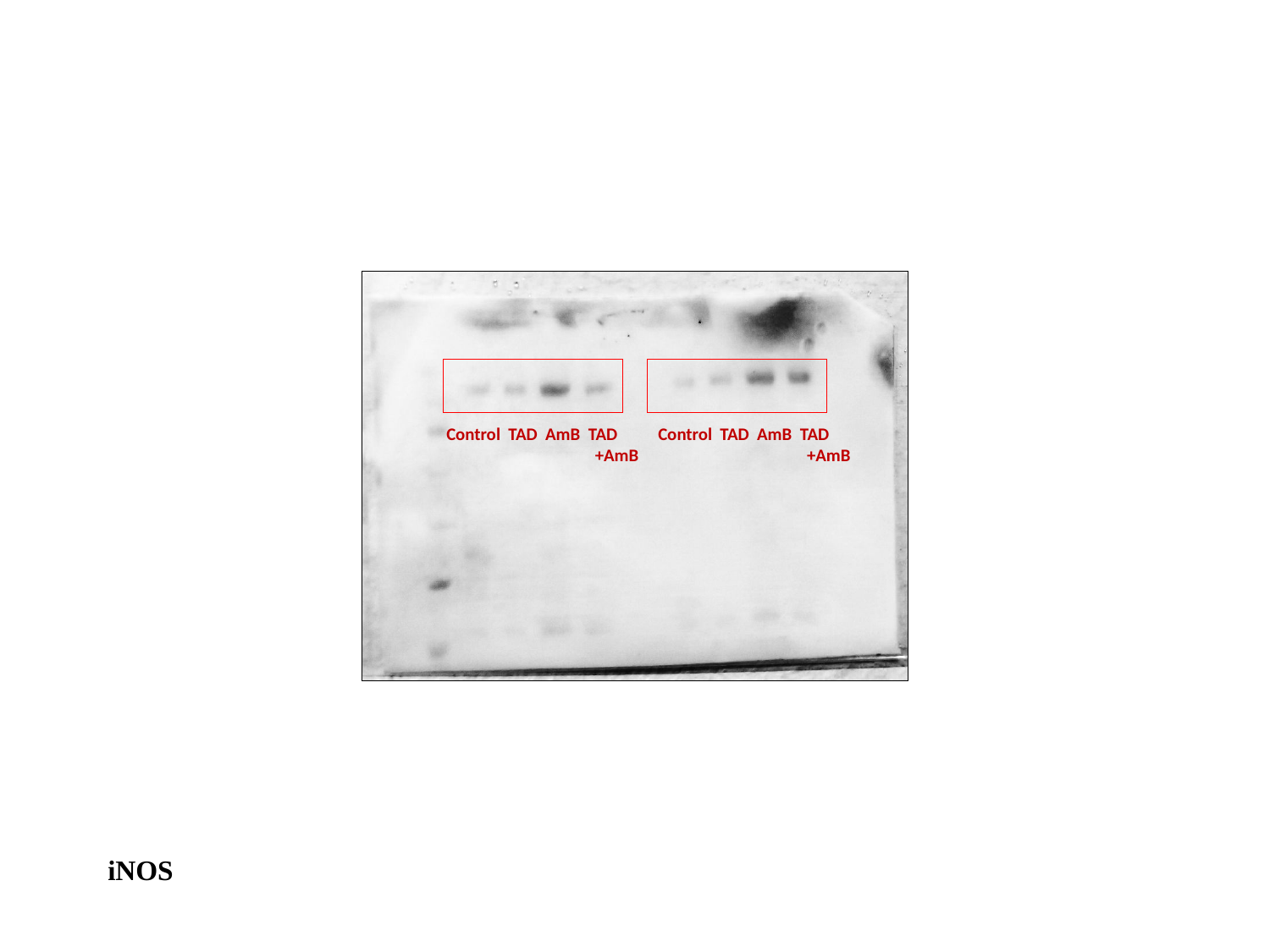

Control TAD AmB TAD
 +AmB
Control TAD AmB TAD
 +AmB
iNOS

## Slide 3
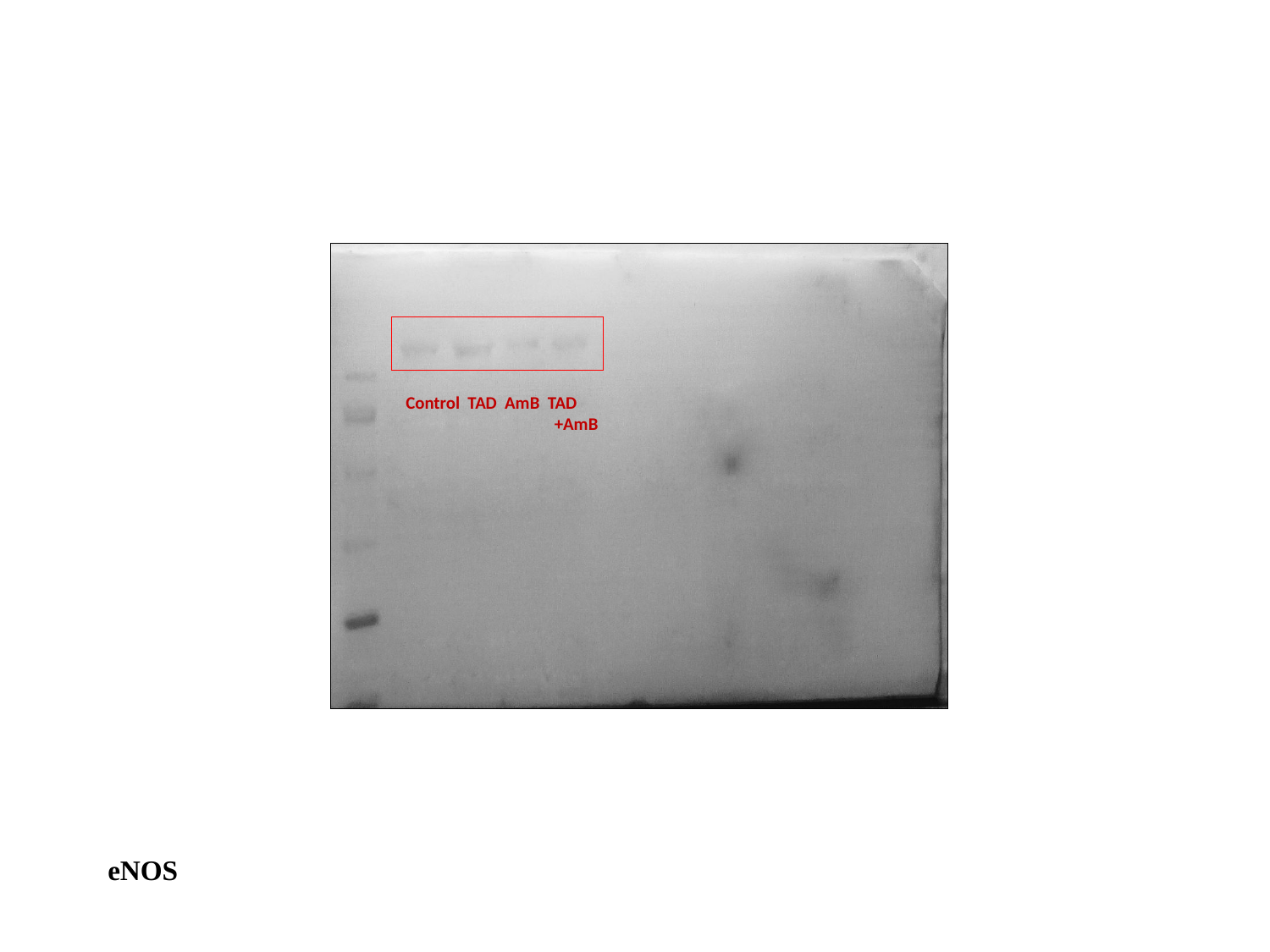

Control TAD AmB TAD
 +AmB
eNOS

## Slide 4
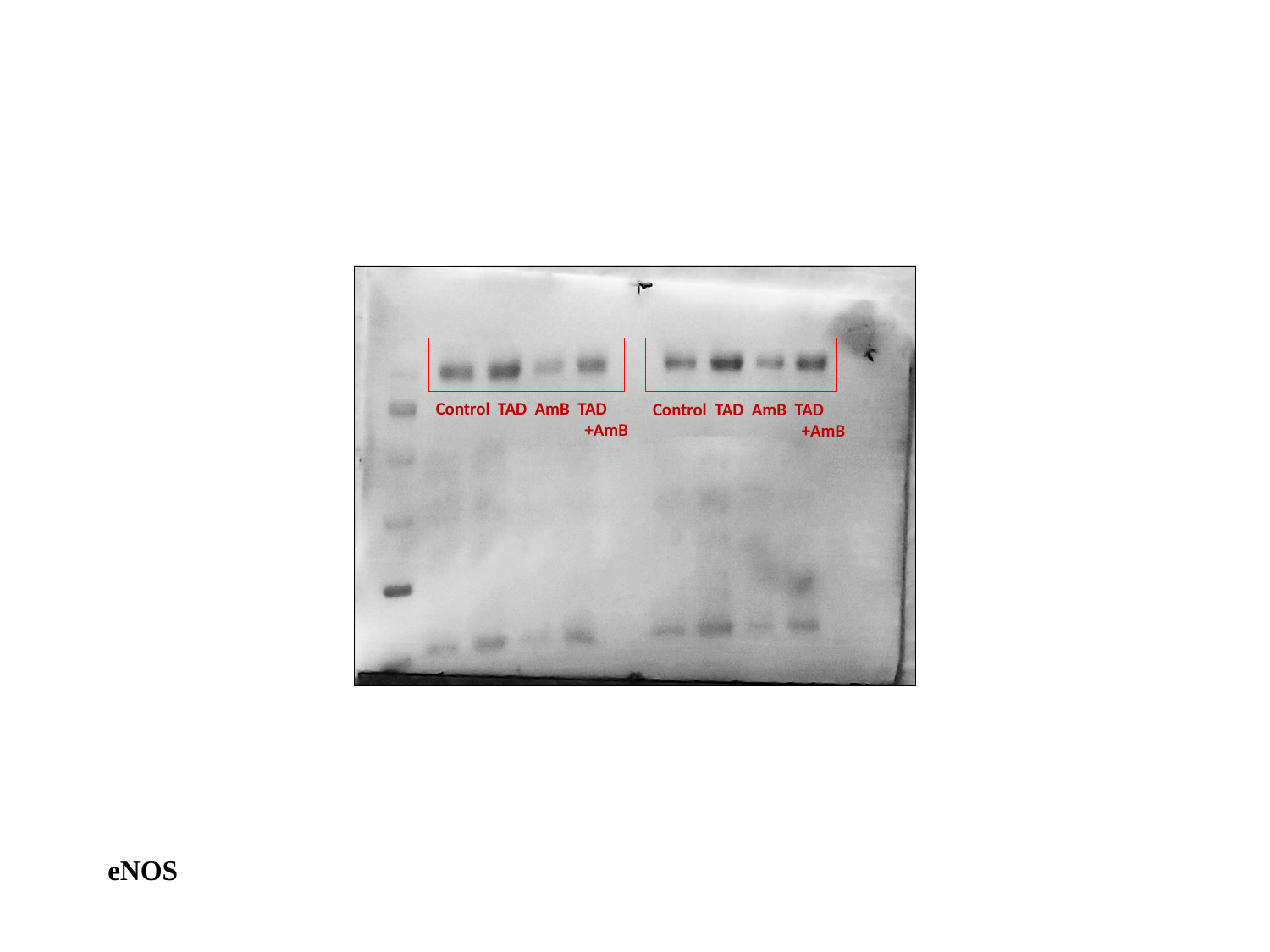

Control TAD AmB TAD
 +AmB
Control TAD AmB TAD
 +AmB
eNOS

## Slide 5
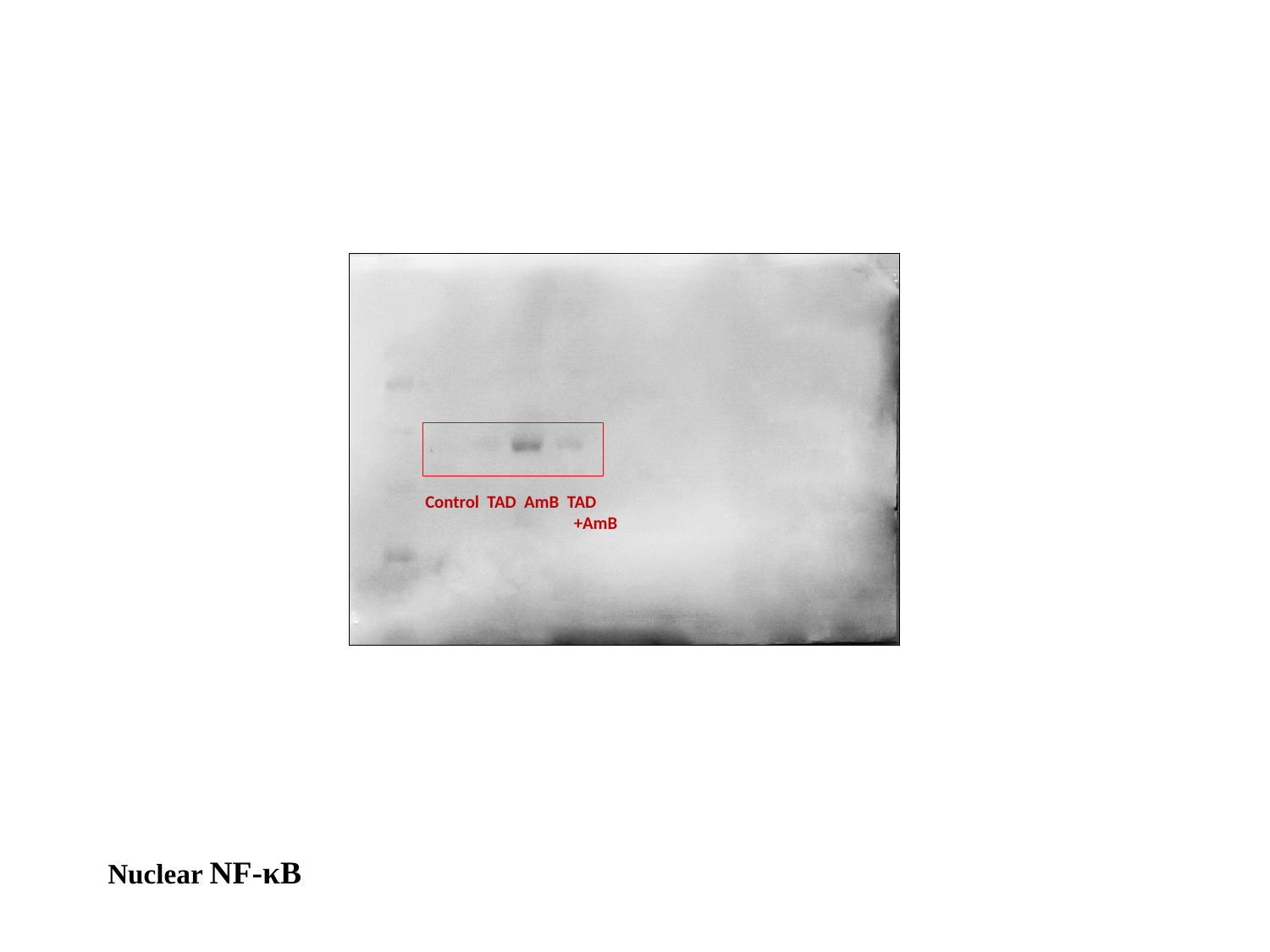

Control TAD AmB TAD
 +AmB
Nuclear NF-κB

## Slide 6
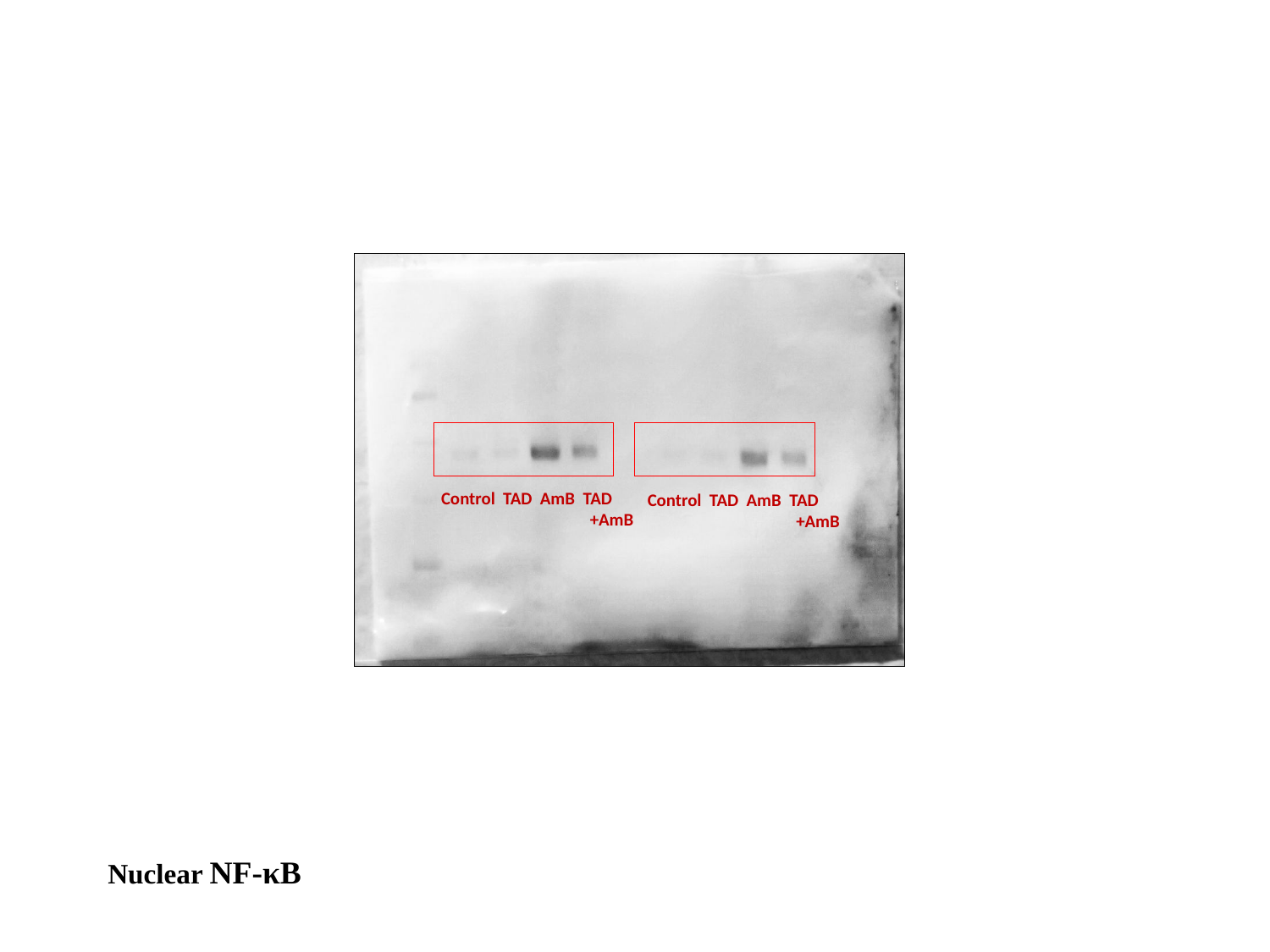

Control TAD AmB TAD
 +AmB
Control TAD AmB TAD
 +AmB
Nuclear NF-κB

## Slide 7
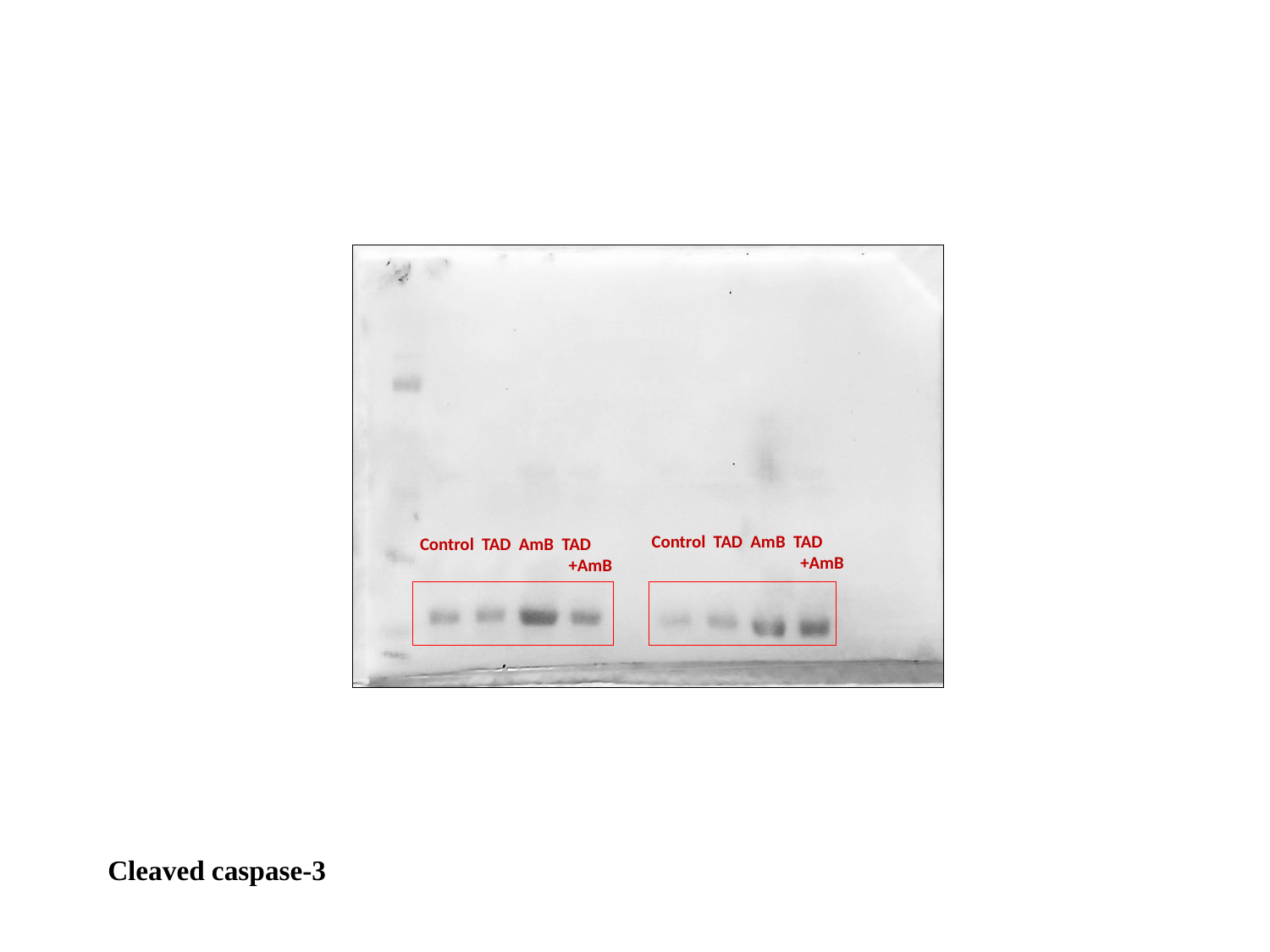

Control TAD AmB TAD
 +AmB
Control TAD AmB TAD
 +AmB
Cleaved caspase-3

## Slide 8
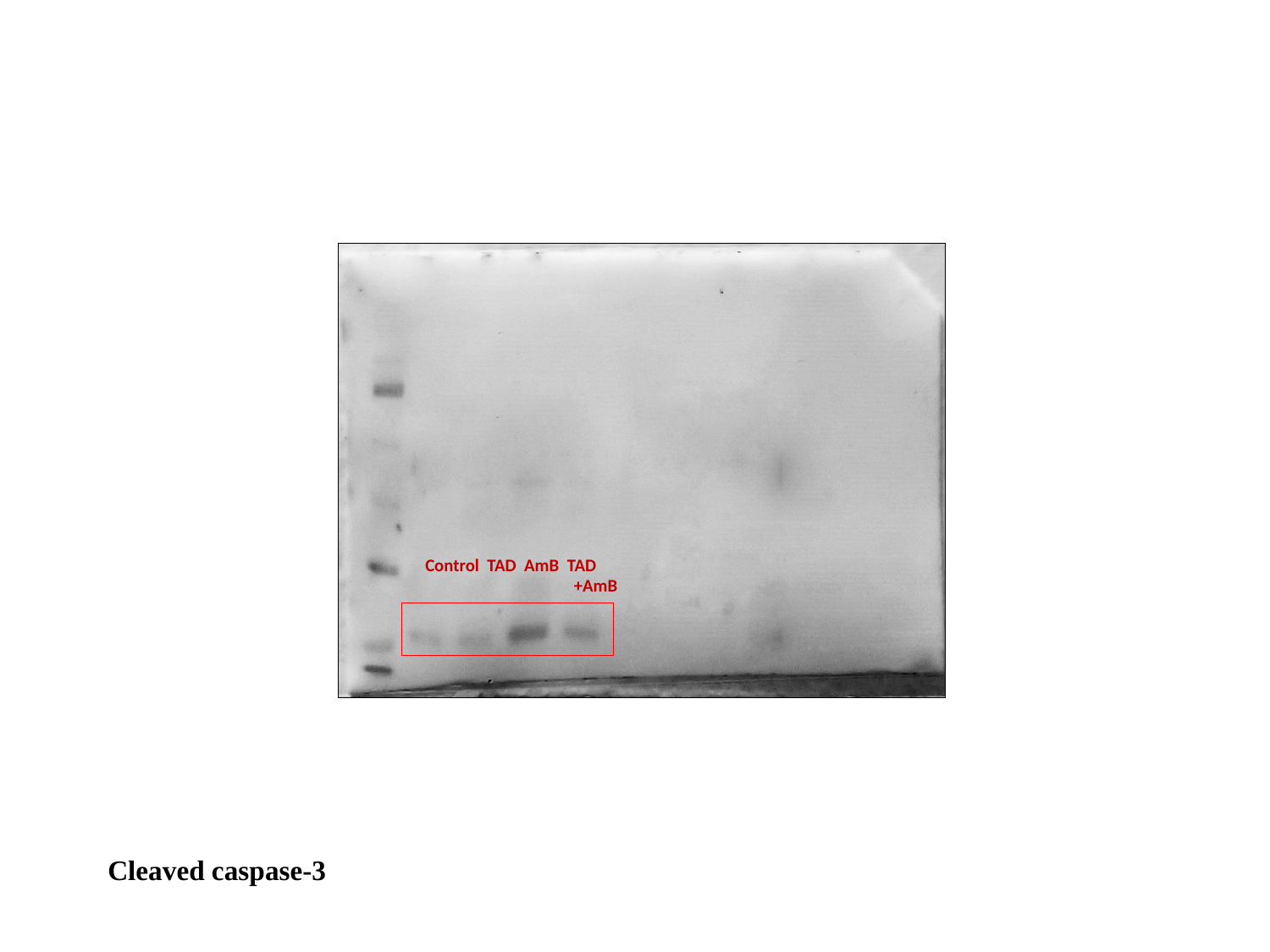

Control TAD AmB TAD
 +AmB
Cleaved caspase-3

## Slide 9
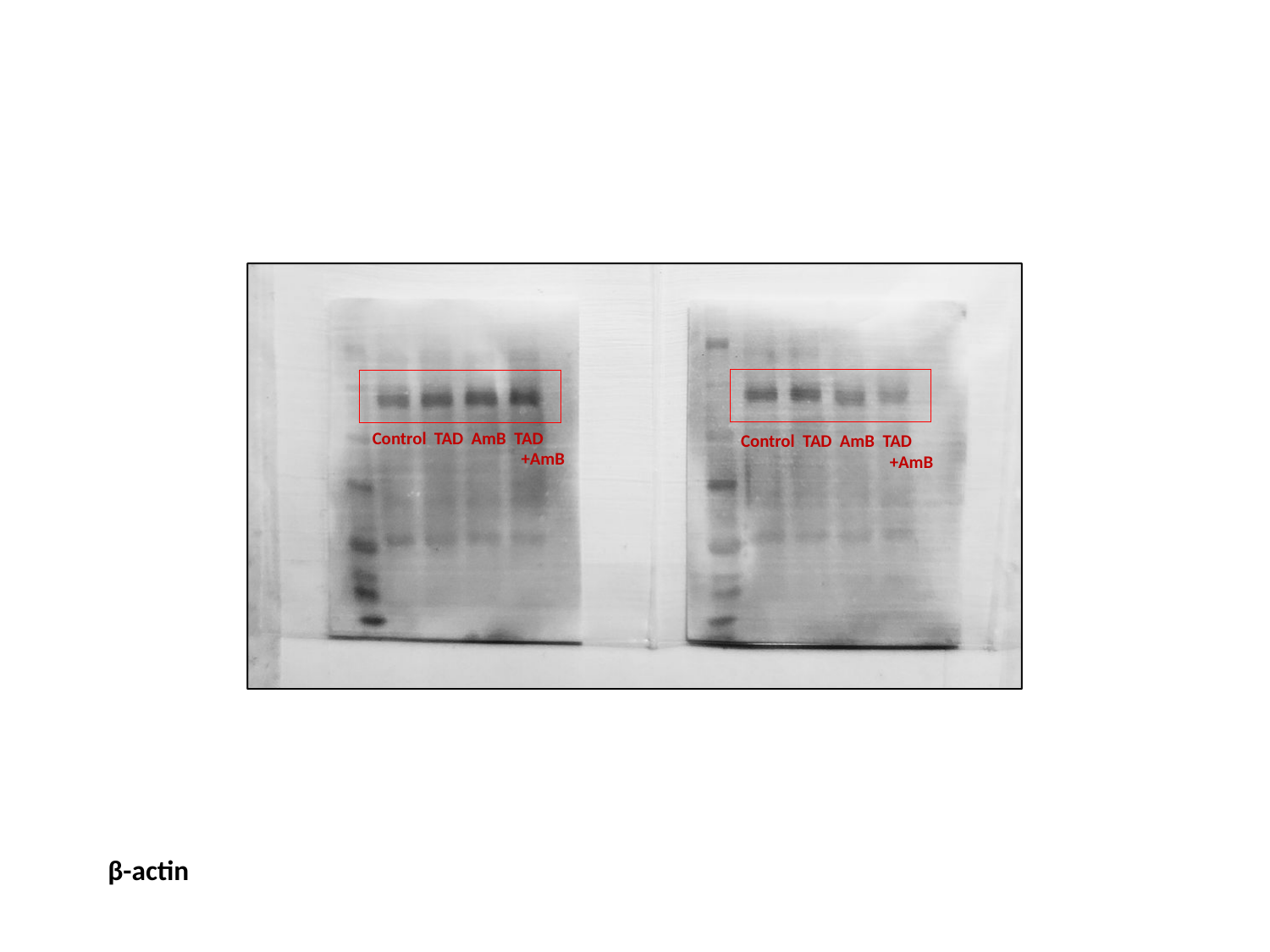

Control TAD AmB TAD
 +AmB
Control TAD AmB TAD
 +AmB
β-actin

## Slide 10
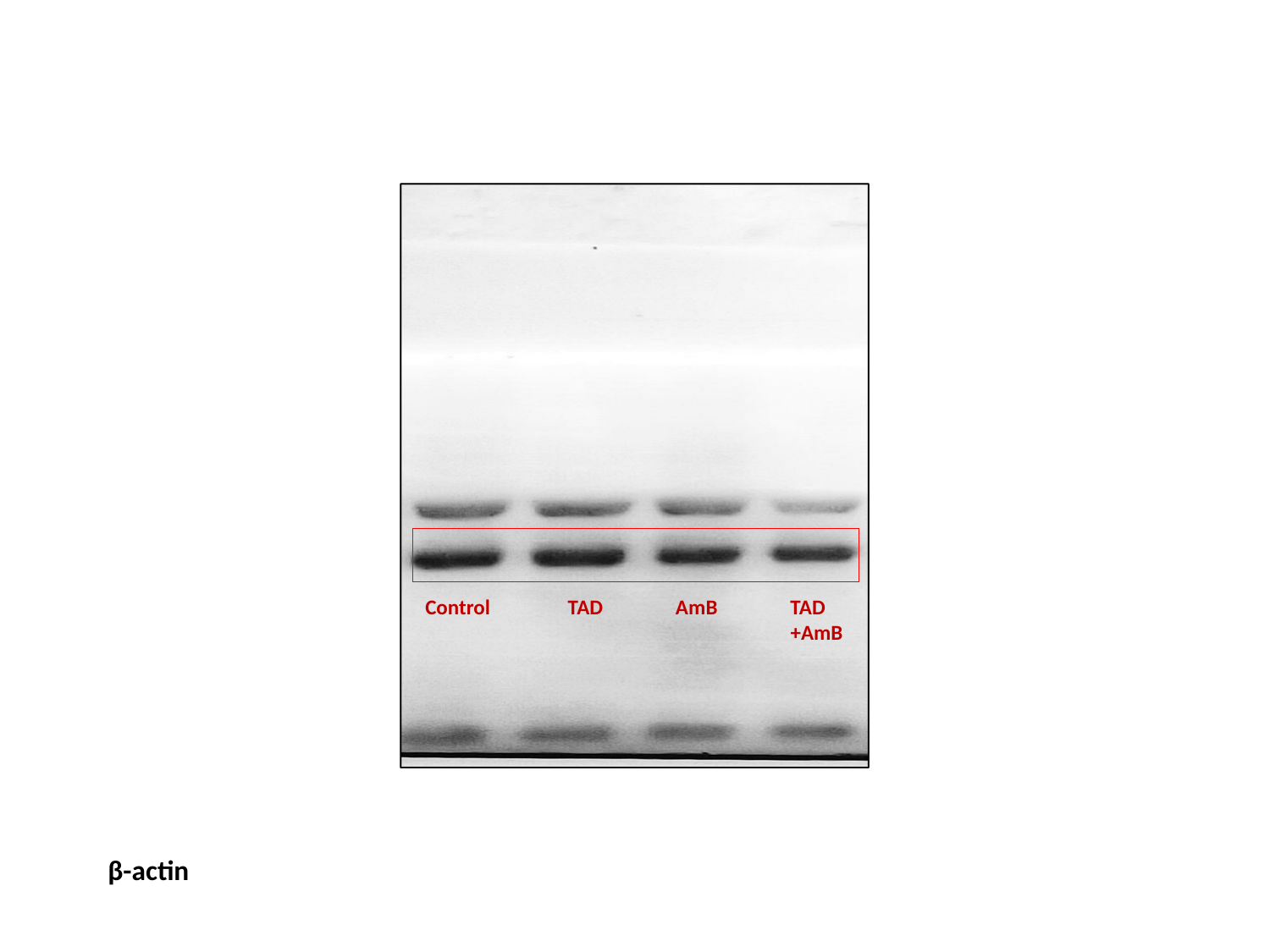

Control TAD AmB TAD
 +AmB
β-actin
